# An Approach to Developing Customized Total Knee Replacement Implants

**DOI:** 10.1155/2017/9298061

**Published:** 2017-11-07

**Authors:** Xinyu Li, Changjiang Wang, Yuan Guo, Weiyi Chen

**Affiliations:** ^1^Institute of Applied Mechanics and Biomedical Engineering, Taiyuan University of Technology, Taiyuan, Shanxi 030024, China; ^2^School of Engineering and Informatics, University of Sussex, Brighton BN1 9QJ, UK

## Abstract

Total knee replacement (TKR) has been performed for patients with end-stage knee joint arthritis to relieve pain and gain functions. Most knee replacement patients can gain satisfactory knee functions; however, the range of motion of the implanted knee is variable. There are many designs of TKR implants; it has been suggested by some researchers that customized implants could offer a better option for patients. Currently, the 3-dimensional knee model of a patient can be created from magnetic resonance imaging (MRI) or computed tomography (CT) data using image processing techniques. The knee models can be used for patient-specific implant design, biomechanical analysis, and creating bone cutting guide blocks. Researchers have developed patient-specific musculoskeletal lower limb model with total knee replacement, and the models can be used to predict muscle forces, joint forces on knee condyles, and wear of tibial polyethylene insert. These available techniques make it feasible to create customized implants for individual patients. Methods and a workflow of creating a customized total knee replacement implant for improving TKR kinematics and functions are discussed and presented in this paper.

## 1. Introduction

Total knee replacement (TKR) has been widely used to relieve osteoarthritis pain, and it has been established as a successful treatment for advanced degenerative joint disease. TKR is expected to rise due to the aging population, obesity, and public expectations. A typical TKR implant has a metal femoral component, a metal tibial tray, a polyethylene insert, and a polyethylene button. One of the main aims of TKR is for a patient to walk postoperatively; however, Milner [1] showed that some patients remain walking abnormally following TKR. The altered gait patterns do not necessarily mean that the TKR has failed, but it may have an impact on the patient's functional capacity in everyday life. For example, more pain, joint stiffness, not able to walk, instability, longer leg, and loose of implanted knee have been reported by patients. Bonnefoy-Mazure et al. [2] presented their research on the evolution of the knee gait kinematic in patients with knee osteoarthritis before and three months after TKR; they pointed out that the disability is still significant for most patients three months after TKR. They suggested that a better understanding of the impairments and functional limitations following surgery would help clinicians design rehabilitation programs. Rahman et al. [3] showed that even 12 months after surgery, many TKR patients have not improved their gait relative to preoperative states. With the abnormal kinematics, the TKR can reduce efficiency of the quadriceps and change patella mechanics, and patients would not have the feeling of a normal knee. The demands in a higher range of motion such as squatting and kneeling require the total knee replacement to provide better function. Lavernia et al. [4] also pointed out that the mean bone mineral density (BMD) in the anterior femoral condylar zone in TKR specimens was significantly lower than that in normal specimens without arthroplasty, most likely due to stress shielding.

In the past decades, there have been attempts to create a more natural feeling and anatomical TKR. The objective of this paper was to review the latest development on TKR, then propose an approach to making customized total knee replacement implants which can function as close as possible to the normal knee of the patient.

### 1.1. Review Methods

Literature review was conducted related to TKR using PUBMED database (US National Library of Medicine and National Institute of Health). There are 336 papers available through a PUBMED search (revised September 25, 2017) using the query “Total knee replacement/arthroplasty” and “patient specific instrumentation.” However, there are 68 papers available through a PUBMED search (revised September 25, 2017) using the query “Total knee replacement/arthroplasty” and “customized.” Among these 68 papers, 28 papers are relevant to the knee replacement and patient-specific instrumentation (PSI) or implant designs. There were recent review papers on PSI by Rodrigues and Gutierres [5] and Alcelik et al. [6]; therefore, this paper will focus on customized TKR implants and musculoskeletal (MSK) modelling of knee joint. Relevant papers searched from ScienceDirect (Elsevier) were also reviewed for the development of customized TKR.

## 2. Design of Total Knee Replacement

**Figure 1:**
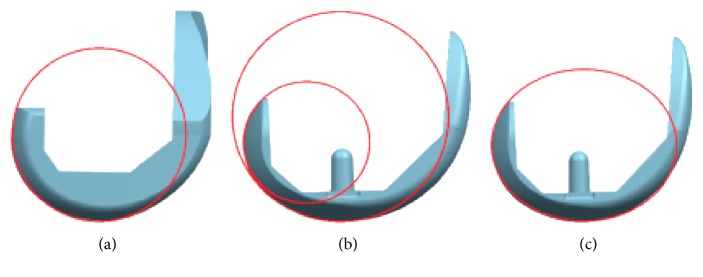
Curvatures of sagittal plane, (a) single circle, (b) multicircle, and (c) ellipse.

**Figure 2:**
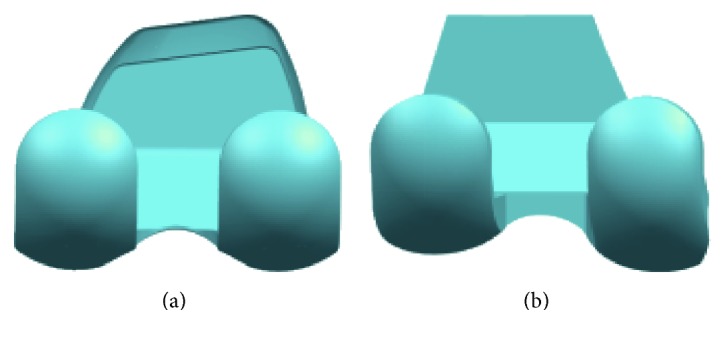
Femoral condyles (a) symmetrical and (b) asymmetrical.

There are many TKR implants available in the market; each of them has its design rationale. Different designs of implant aim to enhance the satisfaction of patients by providing close to normal kinematics. The femoral condyle in sagittal plane may be circular shaped as shown in Figure 1(a) or has multicircles as shown in Figure 1(b), and the J-curve designs are also adopted in femoral component. An oval-shaped design is shown in Figure 1(c). Some of the implants are designed with the same medial and lateral articular surfaces as shown in Figure 2(a); however, asymmetrical articular surfaces have been also designed for achieving close to natural knee kinematics as shown in Figure 2(b).

The motion of the medial compartment in TKR is normally simplified into a ball-and-socket; however, the lateral converged femoral condyle in a surface-guided knee implant has been designed to control the motion of the joint. The lateral condyle may be designed to produce a constant or variable bearing distance between the medial condyle and lateral condyle. To achieve close to normal kinematics in TKR, Walker [7] showed that a knee implant which has medial stability and lateral mobility characteristics should be designed. For example, the SAIPH™ knee (MatOrtho, UK) has been designed to have a medial pivot knee kinematic pattern and an asymmetric posterior translation of the lateral femoral condyle to mimic the natural knee motion. Shimmin et al. [8] studied the stability of the SAIPH knee by videofluoroscopy during four different weight-bearing activities. They concluded that the medially conforming total knee shows a medial pivot motion with tibial internal rotation. However, Warth et al. [9] showed that a medial pivot pattern may not significantly govern clinical success after TKR based on intraoperative kinematics and modern outcome measures. They pointed out that further research is warranted to determine if a particular kinematic pattern promotes optimal clinical outcomes.

Kim et al. [10] compared high-flexion TKR implant with other implants and concluded that there was no improvement with regard to range of motion, clinical outcomes, and the incidence of radiolucent lines despite theoretical range of motion advantages of high-flexion prosthesis. Li et al. [11] studied the kinematics of knee joint with TKR and concluded that the clinical outcome after TKR may be affected by factors such as preoperative range of motion, flexion space balancing, posterior tibiofemoral articular contact stability, and quadricep contraction.

With regard to implant wear, Abdelgaied et al. [12] investigated the effect of tibial insert conformity and material on total knee replacement wear; they concluded that the expected TKR lifetime might be increased by less conforming TKR implant. However, due to the noncongruent and sometimes unstable form of the TKR, wear is a constant issue. Massin [13] reported that wear can be reduced by improving techniques such as choice of implant size, component alignment, and adapted balancing.

Culler et al. [14] compared the outcomes of 126 customized individually made implant and 122 standard off-the-shelf implant patients undergoing TKR. They found that patients treated with customized individually made implant had significantly lower transfusion rates and fewer adverse event rates without increasing costs. White and Ranawat [15] evaluated manipulation rates and clinical outcomes of 21 patient-specific TKRs and off-the-shelf TKRs. They found that the patient-specific knee accurately restored the anatomical joint line and posterior condylar offset; however, patient-specific TKRs were associated with higher manipulation rate and lower satisfaction scores compared to off-the-shelf implants. Research on the comparison of customized cruciate-retaining TKR and asymmetric condylar cruciate-retaining TKA was carried out by Zeller et al. [16], and they concluded that the customized cruciate-retaining TKR demonstrated kinematics more similar to a normal knee.

Many researches showed that women had significantly narrower distal femoral condyle width than men. Wise et al. [17] showed that distal femoral and proximal tibial knee shapes differ by sex and recommended further study to understand the effect of shape modes on the development of osteoarthritis. Li et al. [18] used 3D anatomic models which showed that the shape and the peak positions of anterior condyle groove have gender difference, and they pointed out that the shape of the trochlear groove and the height of medial anterior condyle need to be designed gender specific.

**Figure 3:**
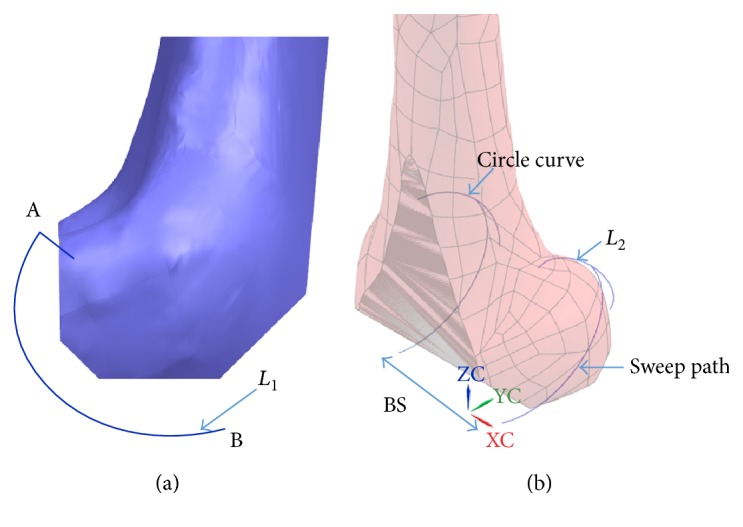
Oval curves derived from the femoral bone model, (a) sagittal view and (b) isometric view.

Customized TKR implant has been developed based on the patient's MRI/CT data.  Figure 3(a) shows a curve *L*_1_ in the sagittal plane; this curve is a condyle profile that matches a patient's knee shape. The circular curve and the other curve *L*_2_ are created based on a patient knee model. The curve *L*_2_ sweeps along the curve *L*_1_ from point A to point B and creates a condylar articular surface. BS is the distance between two condyle profiles, and it is determined from a patient knee model.

Carr and Goswami [19] reviewed knee replacements and biomechanics; they pointed out that issues such as wear and fixation had become more critical with prolonged use of knee implants. Knee implant recipients are more active today than ever; therefore, designing implants that mimic the natural knee is essential to the patients' long-term satisfaction and survival.

## 3. 3D Printed Patient-Specific Instrumentation

**Figure 4:**
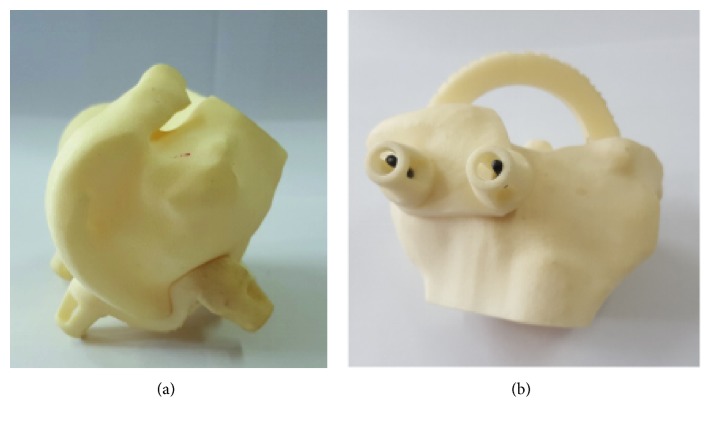
Cutting guide (a) distal femur and (b) tibia.

Lower limb mechanical axis restoration is very important for long-term survivorship of TKR. Recently, patient-specific instrumentation or patient-specific cutting block/guide has been developed to help improve mechanical axis alignment. Medical images can be processed to create 3D models, along with the development of 3D printing technologies. There has been an increased use of 3D printing techniques in patient-specific treatments. 3D printing can be used mostly to create patient-specific anatomical models, customized moulds, surgical guides, and implants. It has been reported that patient-specific guide or cutting block can provide guidance to surgeons during surgery, and this can minimize tissue loss and optimize the positioning of implants. A distal femoral cutting guide is shown in Figure 4(a), and it is used to insert guide pins for cutting block during TKR. A tibial cutting guide is shown in Figure 4(b).

Both MRI and CT imaging have been used for creation of patient-specific guides. MRI is able to account for residual articular cartilage; therefore, the cutting guide can cover a broad contact area and can be directly placed on bone and residual cartilage of knee joint. CT is unable to account for residual cartilage; the corresponding cutting guide has to rely on multiple bony sites. Frye et al. [20] concluded that an MRI-generated template is better than CT-based guides.

Patient knee shapes are well known to be different; the surface geometry of TKR implant affects joint congruence and contact mechanics. It has been suggested by Pati et al. [21] that customized knee replacement from CT scan to 3D printing, customized cutting measures, and customized fitting templates could reduce operation time and assure good alignment. Ganapathi [22] discussed using the technique of patient-specific guides (PSG) to perform TKR; the PSG replaces traditional jigs. To produce PSG, computerized 3D models of the distal femur and proximal tibia are created and the models are used to plan the operation and generate negative moulds of the patient's distal femur and proximal tibia. The operative time may be saved depending on a surgeon's experience and proficiency with the PSG technique. Ganapathi [22] concluded that the advantage of PSG is notable in terms of adequate fit and accuracy of the PSG.

Goyal and Tripathy [23] have surveyed the functional outcomes of total knee replacement using PSI. They pointed out that the PSI is not a patient-matched implant, and the main focus of implant design should be creating the patient-matched implant. Goyal et al. [24] studied the effect of implant design on PSI accuracy, and they concluded that differences in implant design can influence the accuracy of bone resection and component alignment for a given PSI design system.

However, Rodrigues and Gutierres [5] reviewed comparison studies between patient-specific instrumentation (PSI) and standard instruments in TKR, and they noted that PSI had not consistently been shown to be cost-effective or to offer any clinical benefit with regard to functional scores. More studies and longer follow-up period are needed to make definitive conclusions about the PSI efficacy and the potential applicability of PSI to special situations. A similar study by Alcelik et al. [6] showed that PSI is not superior to ST instrumentation in primary total knee arthroplasty.

## 4. Lower Limb Musculoskeletal Model with Total Knee Replacement

Park et al. [25] investigated the relationship of lower limb muscle with pain, function, and frontal plane gait kinematics in patients with osteoarthritis. They confirmed that patients have knee osteoarthritis, reduced hip rotation, knee extension, and ankle inversion strength, but increased peak knee adduction during gait. Also, muscle strength played a significant role in the self-reported function and gait in patient with osteoarthritis.

**Figure 5:**
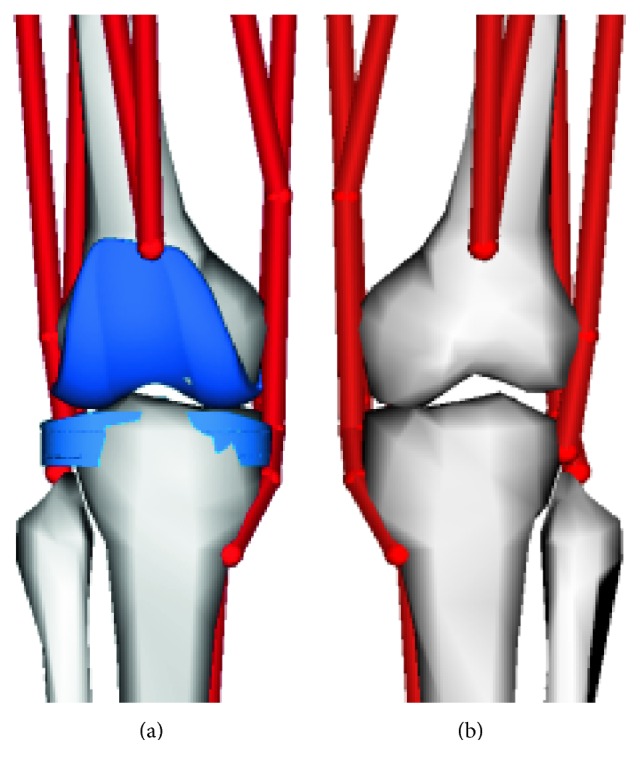
Lower limb musculoskeletal model, (a) knee implant model and (b) knee pin joint model.

Musculoskeletal models can be created using software such as AnyBody and Opensim. A lower limb musculoskeletal model with a TKR implant is shown in Figure 5(a), and it includes main muscles in the lower limb. A normal lower limb musculoskeletal model is shown in Figure 5(b), and the knee joint is simplified as a pin joint that has a rational degree of freedom.

Knowledge of muscle and joint loading is important for evaluating the performance of TKR implant. If the knee is assumed as a pin joint in MSK models, it will produce erroneous results in knee muscle forces and moments acting in the frontal and transverse planes. Walter and Pandy [26] simulated the knee joint articular contact loading during level walking and stair descent, and they integrate a six degree of freedom tibiofemoral joint model into a forward dynamics simulation framework. Medial and lateral tibiofemoral joint contact loads were predicted with good agreement with the experimental data of knee joint loads for level walking.

Lower limb musculoskeletal model has been used in the analysis of joint mechanics and kinematics. Many researchers have tried different methods to create musculoskeletal models. Knarr and Higginson [27] proposed a practical approach to subject-specific estimation of knee joint contact force. A statistical finite element model of knee accounting for shape and alignment variability was developed by Rao et al. [28], and this model can be used to investigate knee joint mechanics and implant design. Belvedere et al. [29] discussed the importance of accurate muscle geometry for musculoskeletal models for subject-specific simulations. They combined a nonlinear scaling technique with a procedure to reconstruct bones from incomplete or scattered geometry data; this method can predict muscle geometries based on bone shapes. During total knee replacement, neutral mechanical alignment is generally targeted. Nolte et al. [30] pointed out that kinematic alignment which is based on the alignment of the prearthritic lower limb can allow better restoration of knee physiological function. Ullrich et al. [31] studied the long-term data of gait characteristics and moment-knee angle relations in female total knee replacement patients, and they found that the patients showed significant gait deficits during constant and self-selected walking speeds and lower average absolute values in the moment-knee angle relations of the knee extensors and flexors. Baldwin et al. [32] developed subject-specific finite element models from imaging data. They demonstrated an integrated approach to facilitate finite element analysis and statistical shape modelling of knee structures.

Musculoskeletal models can be used for implant wear analysis. TKR implant has traditionally been tested in knee wear simulator to determine its ability to resist wear. The computational models can be used to predict wear of implant as did by Zhang et al. [33], and they created a patient-specific wear prediction framework for TKR implant combined musculoskeletal multibody dynamics and finite element analysis. An interesting research was carried out by Chen et al. [34], and they created a full lower limb subject-specific musculoskeletal model that is scaled from a generic MSK model according to patient's CT images and gait dataset. In this model, a total knee replacement implant was modelled. Contact and ligament forces were predicted using a force-dependent kinematics method. This approach is very useful for design-customized TKR implants. Shi et al. [35] used computational models to predict stresses in TKR implant; the model was used to compare the performance of implants. Pejhan et al. [36] evaluated the kinematic performance of a customized surface-guided TKR implant using virtual simulation and load-controlled knee wear simulator. They concluded that virtual simulation is a valid tool for future evaluations of the customized surface-guided TKR implants. Wang et al. [37] evaluated knee strength and mechanics during walking for patients with either a modern off-the-shelf TKR or a customized bicompartmental knee replacement after one year postsurgery. They concluded that the patients with bicompartmental knee replacement exhibit better strength and mechanics while performing daily activities.

Patient's gait dataset was used in the modelling, and this may raise the question of obtaining the gait data. The evolution of the knee gait kinematics in patients with knee osteoarthritis before and several months after a total knee replacement has been researched and presented by Bonnefoy-Mazure et al. [2] and Rahman et al. [3]. Kramers-de Quervain et al. [38] reported that two years after TKR there were significant improvements in gait velocity, cadence, and most of the ground reaction parameters; however, forces during loading and unloading remained lower for the operated leg than for the contralateral leg. Therefore, a patient's two-year postoperative gait could be predicted if a patient's preoperative gait is measured. With the patient's own gait data, a customized TKR implant could be developed.

## 5. Discussion

**Figure 6:**
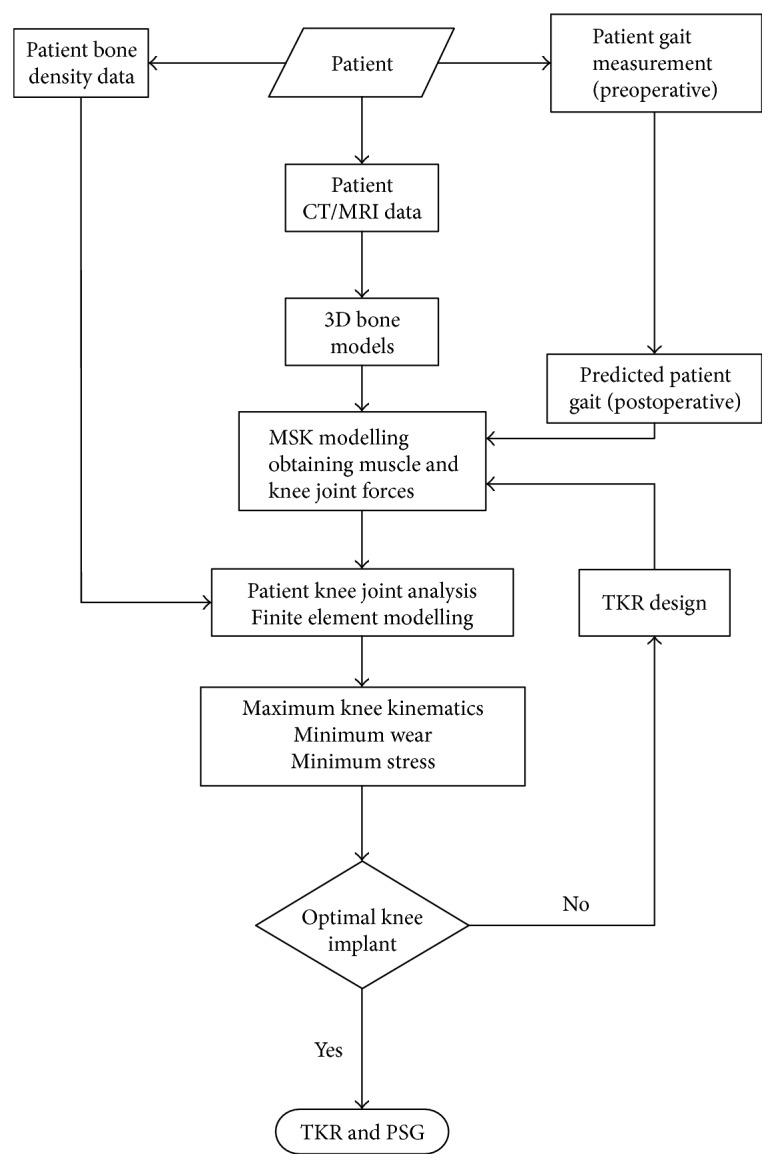
A schematic flow of designing a customized TKR implant.

Based on the progress described in the previous sections, a new approach which is different from the current customized knee implant design is proposed in this paper. The focus of this approach is using patient-specific loading and gait dataset for knee implant designs in addition to the knee joint anatomical features. The procedure of creating a customized TKR implant is shown in Figure 6.

To design a customized TKR implant, 3D computer models from CT scans or MRI should be created firstly. The gait and foot reaction forces of the patient will be measured preoperatively and used for the prediction of load and kinematics of knee joint. Postoperative gait characteristics of patient can be predicted using the derived relationship between measured preoperative and postoperative gait datasets; then, the predicted patient gait is used in the MSK modelling. The interaction between TKR implant and knee joint dynamics will be evaluated using MSK models. The optimal TKR implant should reproduce knee function, maintain bone-implant interface integrity, and resist wear. The kinematics and loads on the TKR implant are very important to the success of TKR. To create a customized TKR implant, an iteration procedure is required to optimise stress, material wear, and knee kinematics.

Customized TKR implant has the potential to greatly improve knee kinematics and patient knee functions compared to off-the-shelf TKR implant. However, further studies need to be carried out to make the customized TKR implant available for patients.

## 6. Conclusion

Customized total knee replacement implant has been previously designed considering knee anatomical shape; however, with the latest development on lower limb musculoskeletal models, force dependent kinematics, and wear simulations, a customized total knee replacement implant could be developed to enhance patient satisfaction. The workflow of the approach to making customized TKR implant is presented in this paper. The customized total knee replacement implant will not only replicate the shape of the knee joint but also to restore normal gait of the patient postoperatively.
